# Failure of Nonoperative Management following Angioembolization for Blunt Splenic and Pancreatic Tail Injury

**DOI:** 10.1155/2020/8863885

**Published:** 2020-10-29

**Authors:** Kazuhiro Nishida, Tadao Kubota

**Affiliations:** Department of General Surgery, Tokyo Bay Medical Center, 3-4-32, Todaijima, Urayasu City Chiba 279-0001, Japan

## Abstract

**Background:**

Over several decades, standard management of blunt spleen injury (BSI) has been changed from operative intervention to the selective operative and nonoperative management (NOM). However, some patient needs laparotomy first. This article describes a case of a BSI patient who failed nonoperative management after angioembolization (AE). *Case Presentation*. A 58-year-old man fell from his motorcycle and was brought to our hospital. His vital sign was stable after extracellular fluid bolus. A contrast-enhanced computed tomography scan of the abdomen showed AAST grade V spleen injury. AE was performed for the splenic artery, but his systolic blood pressure suddenly dropped under 60 mmHg. The resuscitative endovascular balloon occlusion of the aorta was inserted, and immediate laparotomy was performed. A pancreatic tail injury was detected, and the splenic artery and vein were burst at the pancreatic tail and controlled by hemostatic suture. After splenectomy, a drain was placed at the pancreatic tail and the abdomen was temporally closed. The postoperative course was not remarkable except for abdominal abscess treated with antibiotics, and he was discharged on foot.

**Conclusion:**

Although NOM is becoming one of the choices for severe BSI, there will still be a patient who requires surgery. Surgeons should be aware of the mechanism of injury and the limitation of AE as an adjunct to NOM. Patient selection for initial NOM and timing to convert to laparotomy are important.

## 1. Background

Over several decades, standard management of blunt spleen injury (BSI) has been changed from operative intervention to the selective operative and nonoperative management (NOM). Current Eastern Association for the Surgery of Trauma (EAST) guidelines, updated in 2012, recommend NOM for any American Association for the Surgery of Trauma (AAST) grade injury in a hemodynamically stable patient and angioembolization (AE) for patients with AAST grade injuries greater than III, the presence of a contrast blush, moderate hemoperitoneum, or evidence of ongoing splenic bleeding [1]. However, some patient needs laparotomy first. This article describes a case of a BSI patient who failed nonoperative management after AE.

## 2. Case Presentation

**Figure 1:**
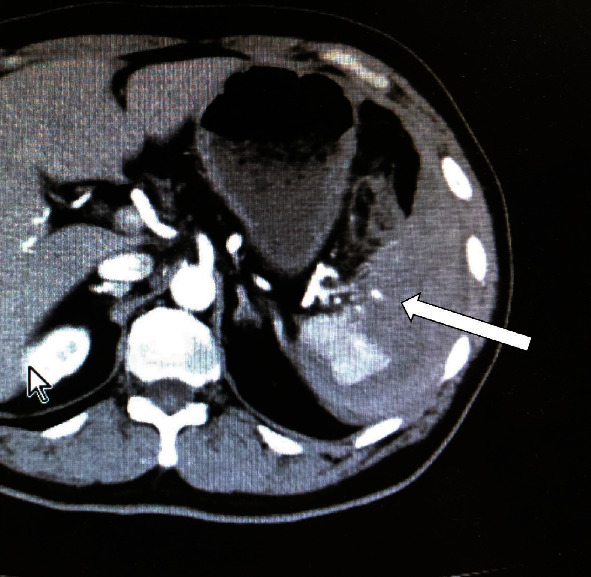
The contrast-enhanced computed tomography (CT) scan of the abdomen showed a shattered spleen and contrast blush (arrow).

A 58-year-old man fell from his motorcycle and was brought to our hospital. His medical history was remarkable for hypertension and cerebral infarction for which he is taking clopidogrel sulfate. His pulse rate was 71 per minute and his blood pressure 75/39 mmHg which raised up to 134/65 mmHg responding to 1000 ml extracellular fluid bolus. There was no body surface injury on inspection, but abdominal ultrasonography revealed a large amount of hematoma surrounding the spleen. His hemoglobin level was 6.2 g/dl without any coagulopathy. A contrast-enhanced computed tomography (CT) scan of the abdomen showed AAST grade V spleen injury (Figure 1). The emergency physician performed AE of the splenic artery and consulted to our department for admission. Although his vital sign was stable, immediate laparotomy was planned for splenectomy and exploration of other organ injuries because he had massive intraperitoneal hemorrhage, and abdominal midline blow, which concerns about pancreatic injury, was suspected from the situation. Before arriving at the operating room (OR), his systolic blood pressure (SBP) suddenly dropped under 60 mmHg despite all efforts of packed red blood cell pumping. The resuscitative endovascular balloon occlusion of the aorta (REBOA) was inserted, and he was transported to the OR. Crash laparotomy was done, and the abdomen was entered. The whole abdomen was explored, and splenic and pancreatic tail injury was detected without any hollow viscous injury. The splenic artery and vein were burst at the pancreatic tail and controlled by hemostatic suture. After splenectomy, a drain was placed at the pancreatic tail and the abdomen was temporally closed. The postoperative course was not remarkable except for abdominal abscess treated with antibiotics, and he was discharged on postoperative day 68.

## 3. Discussion

Generally, the treatment strategy of BSI depends on the AAST grade and hemodynamic stability. Recent literature reported that AE as an adjunct to NOM decreases the NOM failure rate by less than 10% even in grade IV-V injuries [2]. In 2013, Cirocchi et al. performed a systematic review and concluded that they could not clarify the safety and efficacy of NOM for severe splenic trauma because of the selection bias and heterogeneity of the studies [3]. In 2017, Crichton and his colleagues compared the effectiveness of AE as an adjunct to NOM with that of NOM alone in all grade BSI and concluded that AE significantly improves the success of NOM of AAST grade IV and V BSIs [4]. As seen above, once NOM was selected for high-grade BSI, AE should be performed, although safety of NOM for high-grade BSI is controversial.

Hemodynamic status is a very important factor for the initial management of BSI, and there is no doubt that hemodynamically unstable patient needs surgery. World Society of Emergency Surgery (WSES) guidelines, published in 2017, contain hemodynamic status in its classification [5]. According to the guideline, NOM is recommended for the first-line management of stable grade IV-V injuries and this approach is the same as that in EAST guidelines. Of note, both guidelines recommend NOM only in an environment that provides close monitoring with immediate access to surgery. Such environment can easily be achieved in a level 1 trauma center in the US, but it may be difficult in many Japanese community hospitals where the trauma system is not constructed enough.

The definition of hemodynamic stability greatly varies in the literature. Traditionally, shock was defined as SBP lower than 90 mmHg, and recent large database analysis from the US and UK reveals that initial SBP lower than 110 mmHg is associated with increased mortality in trauma patients [ 6, 7]. For this patient, even though he responded to immediate resuscitation, laparotomy should be done from the beginning because his initial SBP was 75 mmHg.

As another factor, the mechanism of injury should be paid more attention. For our patient, abdominal midline blow was strongly suspected because he had no scar on his abdomen and no rib fracture, and it is known as the typical mechanism of pancreatic injury. Besides massive hemoperitoneum, it is also the reason why we planned immediate laparotomy when his hemodynamic status was stabilized after AE. The preoperative diagnosis was solo splenic injury, but pancreatic injury with splenic vein laceration was detected intraoperatively. Thus, NOM is inferior to surgical exploration in diagnosis and AE is not useful for venous hemorrhage. Surgeons should be aware of the limitation of NOM and AE and never miss the timing to open the abdomen.

## 4. Conclusion

Although NOM is becoming one of the choices for severe BSI, there will still be a patient who requires surgery. Surgeons should be aware of the mechanism of injury and the limitation of AE as an adjunct to NOM. Patient selection for initial NOM and timing to convert to laparotomy are important.
